# Effects of 5-Ammonium Valeric Acid Iodide as Additive on Methyl Ammonium Lead Iodide Perovskite Solar Cells

**DOI:** 10.3390/nano10122512

**Published:** 2020-12-14

**Authors:** Daming Zheng, Changheng Tong, Tao Zhu, Yaoguang Rong, Thierry Pauporté

**Affiliations:** 1 Chimie ParisTech, PSL Research University, CNRS, Institut de Recherche de Chimie Paris (IRCP), UMR8247, 11 rue P. et M. Curie, F-75005 Paris, France; daming.zheng@chimieparistech.psl.eu (D.Z.); tch13986581570@gmail.com (C.T.); tao.zhu@chimieparistech.psl.eu (T.Z.); 2 Wuhan National Laboratory for Optoelectronics, China-EU Institute for Clean and Renewable Energy, Huazhong University of Science & Technology (HUST), Wuhan 430074, China; ygrong@hust.edu.cn

**Keywords:** perovskite solar cells, 5-ammonium valeric acid iodide, triple mesoscopic solar cells, stability, electrical impedance spectroscopy, charges recombination

## Abstract

During the past decade, the power conversion efficiency (PCE) of perovskite solar cells (PSCs) has risen rapidly, and it now approaches the record for single crystal silicon solar cells. However, these devices still suffer from a problem of stability. To improve PSC stability, two approaches have been notably developed: the use of additives and/or post-treatments that can strengthen perovskite structures and the use of a nontypical architecture where three mesoporous layers, including a porous carbon backcontact without hole transporting layer, are employed. This paper focuses on 5-ammonium valeric acid iodide (5-AVAI or AVA) as an additive in methylammonium lead iodide (MAPI). By combining scanning electron microscopy (SEM), X-ray diffraction (XRD), time-resolved photoluminescence (TRPL), current–voltage measurements, ideality factor determination, and in-depth electrical impedance spectroscopy (EIS) investigations on various layers stacks structures, we discriminated the effects of a mesoscopic scaffold and an AVA additive. The AVA additive was found to decrease the bulk defects in perovskite (PVK) and boost the PVK resistance to moisture. The triple mesoporous structure was detrimental for the defects, but it improved the stability against humidity. On standard architecture, the PCE is 16.9% with the AVA additive instead of 18.1% for the control. A high stability of TiO_2_/ZrO_2_/carbon/perovskite cells was found due to both AVA and the protection by the all-inorganic scaffold. These cells achieved a PCE of 14.4% in the present work.

## 1. Introduction

During the past decade, organic–inorganic halide perovskites (PVKs) have risen as one of the most promising semiconductor families for various advanced applications in optoelectronics, such as light emitting diodes (LEDs) [1], lasers [1], photodetectors [2], scintillators [3], and solar cells [4,5,6,7,8,9,10,11,12,13,14,15,16,17,18,19,20,21,22,23,24,25,26,27,28,29,30,31,32,33,34,35,36,37,38,39,40,41,42,43]. PVKs are especially promising for photovoltaic applications due to a broad range of favorable properties: (i) preparation from solutions, at low temperature and low cost, (ii) long charges diffusion lengths, (iii) direct optical transition, (iv) a bandgap that can be tuned by playing on the material composition, and (v) low exciton binding energy [2,3,4,5,6,7,8,9,10,11,12,13,14,15,16,17,18,19,20]. As a result of a huge and impressive research effort from the international scientific community, solar cells based on PVKs, called perovskite solar cells (PSCs), have reached a present record efficiency of 25.5% [21]. This places the PSC technology as the most efficient of the thin films ones and close to the best crystalline silicon performances. However, PSCs suffer from several drawbacks, the main bottleneck being the limited stability.

In its classical architecture, a PSC is composed of an organo-lead halide perovskite layer that acts as the solar light absorber and two selective contacts: A hole-transporting layer (HTL) is placed on the top of the PVK to collect and transport hole charge carriers and block electrons, and on the other side, an electron transporting layer (ETL) that collects and transports electrons and blocks the photogenerated holes is placed. To date, 2,2′,7,7′-tetrakis(*N*,*N*’-di-p-methoxyphenylamine)-9,9′-spirobifluorene (Spiro-OMeTAD) is the most popular hole-transporting material (HTM), and TiO_2_ is a popular electron-transporting material (ETM). However, a different cell architecture has proven more suitable for producing highly stable devices [22,23,24,25,26,27,28,29]. It is composed of three stacked mesoporous layers, i.e., TiO_2_/ZrO_2_/carbon. This porous stack is subsequently filled by the perovskite material using a drop-casting technique. In this architecture, no HTM is employed. ZrO_2_ acts as an insulating layer that prevents the direct contact of the carbon backcontact with the TiO_2_ front selective contact. Aside from the high stability, this type of cell architecture is compatible with screen-printing techniques and could enable a low-cost production of PSC panels at a large scale [22,27,28]. Mei et al. were the first to develop these triple-mesocopic solar cells [22]. In their pioneering work, they observed that 5-ammonium valeric acid iodide (HOOC(CH_2_)_4_NH_3_I, noted as 5-AVAI or AVA) was an important additive for getting a good pore filling and a more complete contact of PVK with the mesoporous TiO_2_ scaffold. They stressed that the carbon top electrode presents hydrophobic properties and acts as a barrier against moisture. Later, Grancini et al. [23] showed that AVA allows for the dimensional engineering of the perovskite and the formation of a gradually-organized multidimensional interface. These authors achieved a 12.9% PCE with laboratory cells. They also prepared mini-modules that demonstrated a 11.2% efficiency, which is stable for more than 10,000 h measured under AM 1.5 G standard condition, at 55 °C, and short circuit. By employing slot-die coating of AVA-MAPI, one of us and the coworkers achieved a PCE of 12.9% on the triple-mesoscopic mini-modules of 60 cm^2^ [28]. Very recently, these PSCs successfully passed the main items of IEC61215:2016 PV qualification tests, including the damp heat test, thermal cycling test, and ultraviolet preconditioning test, and they exhibited over a 9000-h operational stability [29].

AVA is the classical additive employed in triple mesoscopic solar cells [22,23,24,25,26,29] even when the 4-(aminomethyl) benzoic acid hydroiodide has also been shown to be very efficient [24]. AVA would play an active role in the high stability of PSCs due to surface defect passivation [30]. Recently, Péan et al. [31] showed that methylammonium (MA^+^) lead triiodide (MAPI) modified by AVA reduces the generation of superoxide when infiltrated in the triple-mesoporous layer stack. Its optimum content in the perovskite precursor solution is reported at 3–4% in the literature [23,24]. An AVA additive was also employed in lead-free PSCs [32]. It was shown to affect the growth of perovskite crystals based on formamidinium and tin. The additive plays on the crystal growth through hydrogen bond with I^−^ and a precursor SnI_6_^4−^ octahedral. It forms a protective layer and acts as a cross-linker at the grain boundaries. Consequently, this additive dramatically improves the performance of tin-based PSCs [32]. AVA was employed recently to post-treat MAPbI_3_ films that allowed for a significant improvement of the solar cell performances and the stabilization of the PVK [33].

The aim of the present paper is to investigate the effect of an AVA additive on PSCs with two different architectures: a structure employing a single TiO_2_ mesoporous layer (noted as *1mp*) and another one employing the (TiO_2_/ZrO_2_/C) triple-mesoporous-layer structure (noted as *3mp*). The effects of the AVA additive and of the host structure on the PVK properties and stability against moisture are first established. Then, the performances of the cells are characterized. We subsequently study the effect of light intensity on the cell electrical response at open-circuit voltage (*V_oc_*) prior to investigate the effects of the applied voltage (*V_appl_*). The influence of the AVA additive and cell architecture on the electrical impedance response of the devices is thoroughly reported and analyzed.

## 2. Materials and Methods

*1mp* substrate preparation: The fluorine-doped SnO_2_ (FTO) substrates (TEC 7 NSG, Tokyo, Japan) were prepared as described in our previous work in Ref. [37]. A compact TiO_2_ layer was first deposited by spray pyrolysis, and a mesoporous TiO_2_ layer was prepared by spin-coating as described in our previous works [16,37,38].

*1mp* cells preparation: The MAPI precursor solution was prepared by dissolving PbI_2_ (TCI) and MAI (Greatcell, Queanbeyan, Australia) in anhydrous DMSO (Sigma-Aldrich, Saint-Quentin Fallavier, France). The double-cation-based perovskite (noted as AVA-MAPI), with the (AVA)_2_MA_56_Pb_57_I_172_ general formula, was prepared by dissolving PbI_2_, MAI, and 5-ammonium valeric acid iodide (TCI) (HOOC–(CH_2_)_4_–NH_3_I) (3.5% molar ratio of PbI_2_) in anhydrous DMSO and stirred at 100 °C for 1 h. For both the MAPbI_3_ and (AVA)_2_MA_56_Pb_57_I_172_ precursor solutions, the concentration of PbI_2_ was 1.45 M. These solutions were spin-coated on the *1mp* substrate using a two-step program at 1000 rpm and 6000 rpm for 10 and 30 s, respectively. In addition, 100 μL of chlorobenzene was dropped 30 s after starting the spinning routine. The films were subsequently annealed at 100 °C for 1 h. The hole transporting material (HTM) was 2,2′,7,7′-tetrakis(N,N’-di-p-methoxyphenylamine)-9,9′-spirobifluorene (Spiro-OMeTAD from Borun New Material Technology, Ningbo, China), which was prepared as detailed in [35]. The gold backcontact was deposited by thermal evaporation through a mask. The pure 2D perovskite (AVA)_2_PbI_4_ layer was prepared by dissolving PbI_2_ and 5-ammonium valeric acid iodide in a 1/2 molar ratio in anhydrous DMSO and stirring at room temperature. The concentration of PbI_2_ was 1.20 M.

*3mp cells preparation*: For the triple mesoscopic devices, the PbI_2_ and γ-Butyrolactone (GBL) were purchased from Sigma-Aldrich, Saint-Quentin Fallavier, France. The methylammonium iodide (MAI) and 5-ammonium valeric acid iodide were synthesized as previously reported [7,22]. The perovskite precursor solution was prepared by dissolving 0.461 g PbI_2_, 0.153 g MAI, and 0.0086 g 5-AVAI in 1.17 mL GBL. It was stirred at 60 °C overnight before use. The TiO_2_ paste was purchased from Greatcell Solar, Queanbeyan, Australia (30 NR-D). The ZrO_2_ paste and carbon paste were prepared as described in [39]. Unless stated otherwise, all the materials were used as received without further purification. The FTO conducting glass substrates were first etched with a 1064 nm laser and then cleaned by ultrasonication with detergent solution, deionized water, and ethanol for 15 min, respectively. A compact TiO_2_ layer was deposited on the glass/FTO substrates by spray pyrolysis deposition at 450 °C using a disopropoxytitaniumbis(acetyl acetonate) solution. Then, the mesoporous TiO_2_ layer, the ZrO_2_ spacer layer, and the carbon layer were layer by layer screen-printed onto the substrates. The TiO_2_ layer and ZrO_2_ layer were sintered at 500 °C for 30 min, and the carbon layer was sintered at 400 °C for 30 min. After it cooled down to room temperature, a 4–4.5 μL of perovskite precursor solution was drop-casted on the top of the carbon layer. After the precursor penetration into the mesoporous scaffold, the samples were annealed at 50 °C for 4 h.

The layers and solar cells were characterized as already described in our recent works [20,40]. The impedance spectra were measured between 600 kHz and 20 mHz with a PGSTAT 12 system from Autolab (Villebon Courtaboeuf, France). The AC signal was 20 mV. The cells were unencapsulated and illuminated with a halogen Schott lamp (Colombes, France) equipped with an optical fiber light guide. Experiments were conducted by either changing the power of the lamp to vary the light intensity from 8 sun% to 90 sun% or by applying a constant voltage (*V_appl_*) between 0 V and the *V_oc_* under 90 sun% illumination. The cell illuminated area was delimited by a 0.16 cm^2^ mask. The full EIS characterization measurements of a PSC lasted typically 1 h. The spectra were analyzed using the Z-view software from National Instrument.

## 3. Results and Discussion

**Figure 1:**
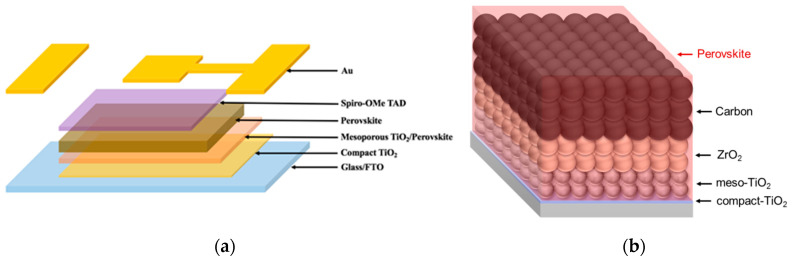
Schematic figures of (**a**) single-mesoporous (*1mp*) and (**b**) carbon-based triple-mesoporous (*3mp*) device structures.

Before developing the characterizations of the PVK layers, one must first precisely determine the two architectures of PSC that have been considered in the present work. As shown in Figure 1, perovskite layers were prepared either by deposition on a mesoporous TiO_2_ layer (Figure 1a, hereafter named *1mp*) or by infiltrating the perovskite precursor solution in a triple mesoporous layers stack combining TiO_2_, ZrO_2_, and carbon. The MAPI perovskite was formed into the pores after an annealing step at 50 °C in an oven (Figure 1b, hereafter named *3mp*). The former corresponds to the standard cell structure where a HTL, made of LiTFSI-doped Spiro-OMeTAD, is deposited on top of the PVK prior to evaporate the gold metal backcontact through a mask to obtain the device. For the latter, the perovskite layer preparation was the final step. The *3mp* cells are HTL-free. For the sake of comparison, we also prepared *1mp* cells without the HTM layer (NoHTM), the gold backcontact being directly evaporated onto the perovskite layer.

### 3.1. Effect of AVA Additive and Substrate on the MAPI Layers Properties

**Figure 2:**
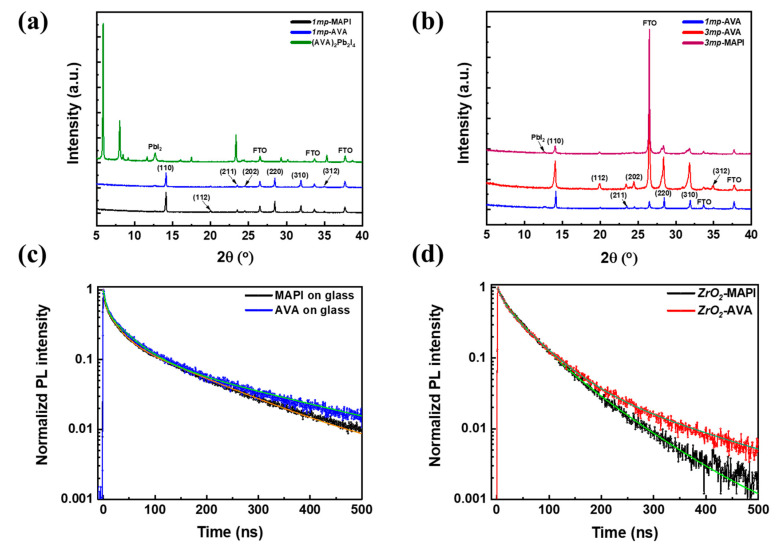
(**a**) XRD patterns of MAPI, AVA-MAPI, and (AVA)_2_PbI_4_ perovskite layers deposited on the *1mp* substrate. (**b**) XRD patterns of *3mp*-MAPI and AVA-MAPI layers deposited on *1mp* and *3mp* substrates. (**c**) TRPL of *1mp*-MAPI and *1mp*-AVA layers deposited on glass substrate. (**d**) TRPL of MAPI and AVA-MAPI layers deposited on mesoporous ZrO_2_/glass.

The effect of an AVA additive on the structural properties of the MAPI layer was investigated by X-ray diffraction (XRD) measurements. Figure 2a compares the MAPI films prepared on *1mp* substrate with and without 3.5% of the AVA additive. The amount of 3.5% was chosen as being among the best in the literature for performances of *3mp* cells [22]. Figure 2a shows that both patterns are dominated by peaks at 14.15° and 28.21°. They correspond to the (110) and (220) planes of tetragonal MAPI and indicate a clear texturization of the prepared layers. The peaks are slightly less intense with the AVA additive. In both cases, a small PbI_2_ diffraction peak at 12.7° is found. In Figure 2a, the XRD pattern of the 2D (AVA)_2_PbI_4_ phase is also presented. Its comparison with the AVA-MAPI pattern exhibits that no peak of the 2D phase is present, so this phase is not present in a significant amount in the final layer. Figure 2b compares the XRD patterns of *1mp*-AVA, *3mp*-AVA, and *3mp*-MAPI layers. We observed the absence of a 2D phase in the first two samples and the absence of PbI_2_ in the *3mp-*AVA sample. The time-resolved photoluminescence (TRPL) curves of MAPI and AVA-MAPI layers deposited on glass and on the ZrO_2_ mesoporous layer are shown in Figure 2c,d and Figure S1a,b (Supplementary Materials). The decay was not monoexponential and a triple exponential function was employed to get a correct fit. The presence of at least three time-constants shows the occurrence of several deexcitation pathways. The longest one (*τ*_slow_) was assigned to the radiative bulk component [20]. The fitting parameters are gathered in Table S1 (Supplementary Materials). In Figure 2c, for layers deposited on glass (an insulating substrate for which charge carrier transfer cannot occur), *τ*_slow_ was measured at 154 ns for MAPI and 227 ns for the AVA-MAPI layers. This component reflects the structural quality of the material which is then improved in the presence of AVA. This trend was confirmed for PVK layers deposited in the ZrO_2_ scaffold (Figure 2d) with a *τ*_slow_ increase from 88 to 181 ns in the presence of AVA additive. If we suppose that the structural quality of perovskites deposited on the *1mp* is close to that deposited on glass, their structural quality is better than the ones prepared in the ZrO_2_ scaffold. 

**Figure 3:**
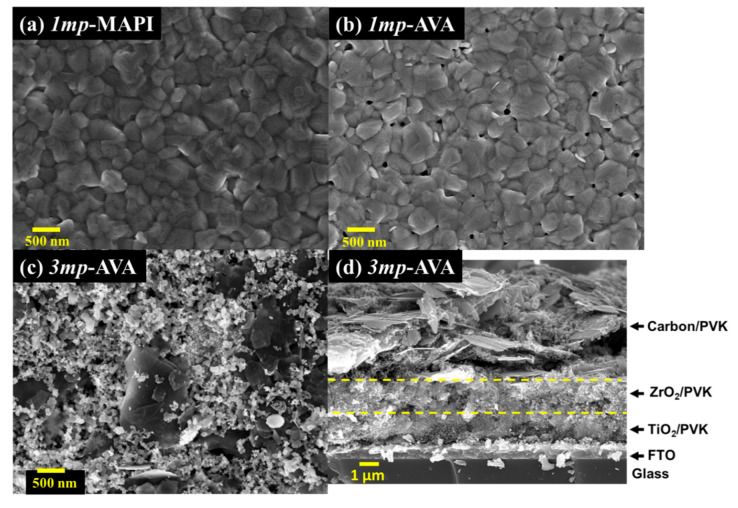
(**a**–**c**) SEM top views of the (**a**) *1mp*-MAPI layer, (**b**) *1mp*-AVA layer, and (**c**) *3mp*-AVA layer. The scale bar is 500 nm. (**d**) Cross-sectional view of the *3mp*-AVA solar cell. The yellow dashed lines visualize the limits between the various mesoporous layers.

The surface morphology of the films was characterized by scanning electron microscopy (SEM). Top views in Figure 3 show that the *1mp* layer was filled and capped by the PVK material. The capping PVK layer presented fewer pinholes for MAPI compared to AVA-MAPI. The average grain size was measured at 320 nm for MAPI and 330 nm for AVA-MAPI. The top aspect was different in the case of the *3mp* substrate (Figure 3c). We observed mesoscopic carbon and graphite flakes. AVA-MAPI filled the mesoporous layers stack, and the top layer was not capped by the PVK material. Figure 3d is a cross-sectional view of the *3mp*-cell where one can recognize the triple mesoporous stack which contains the PVK as schematized in Figure 1b.

**Figure 4:**
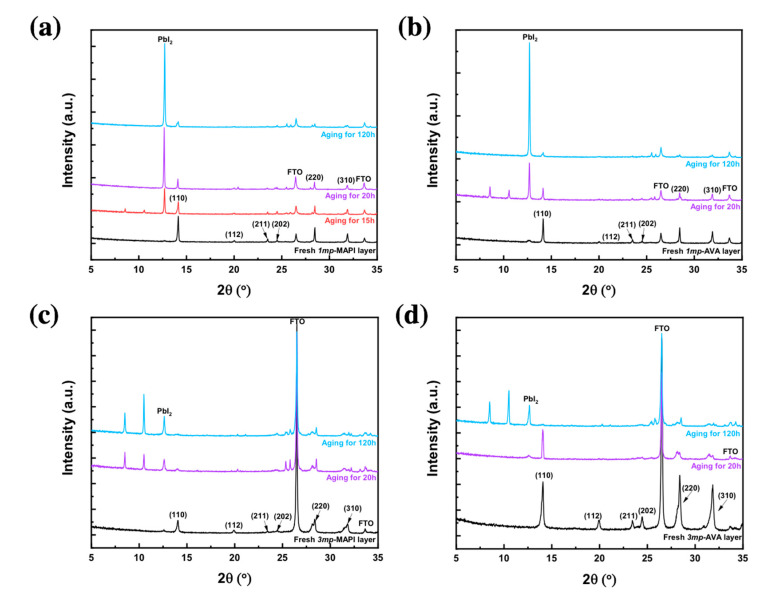
XRD pattern evolution of (**a**) *1mp*-MAPI, (**b**) *1mp*-AVA, (**c**) *3mp*-MAPI, and (**d**) *3mp*-AVA layers upon aging in a 90% RH atmosphere.

We followed the stability of the layers exposed to high humidity (relative humidity (RH) ≥ 90%) at room temperature. To discriminate the effect of AVA and scaffold on PVK stability, we compared the aging of *1mp*-MAPI, *1mp*-AVA, *3mp*-MAPI, and *3mp*-AVA. The phase degradation kinetic was followed by XRD in Figure 4. We can see that, in all cases, the decrease of the PVK diffraction peaks was accompanied by the appearance of three other peaks. The ones at 8.58° and 10.6° are assigned to the monohydrate, the MAPbI_3_∙H_2_O phase [43], and the peak at 12.7° corresponds to PbI_2_. It clearly shows that, under high moisture, MAPI forms a monohydrate species that further decomposes into PbI_2_ while MA is lost. We never observed the di-hydrate phase [43]. The *1mp*-MAPI decomposed rapidly since after 15 h, the pattern was dominated by the hydrate and PbI_2_ phases (Figure 4a). The layer degradation was slower for the *1mp*-AVA sample (Figure 4b) since, after 20 h, the perovskite and hydrate phases were still present when the *1mp*-MAPI XRD pattern was dominated by the PbI_2_ phase. After 120 h, both layers were almost fully degraded into PbI_2_. For the *3mp* samples (Figure 4c,d), we also observed a reduction of the degradation kinetic with the presence of AVA. Moreover, we observed that the *3mp* scaffold is also beneficial for the stability since the hydrate species was still present after 120 h of exposure to moisture while this phase completely disappeared from the *1mp* samples. These observations were confirmed by the layers’ aspect change with aging as displayed in Figure S2 (Supplementary Materials). We can conclude that the high stability of *3mp* cells (TiO_2_/ZrO_2_/Carbon/Perovskite) is mainly due both to the protection by the all-inorganic scaffold and to AVA. AVA can tune the crystallization of the perovskite in the scaffold leading to crystals with less bulk defects.

### 3.2. Effect of AVA Additive and Cell Structure on the Current-Voltage Curves and Performances

**Figure 5:**
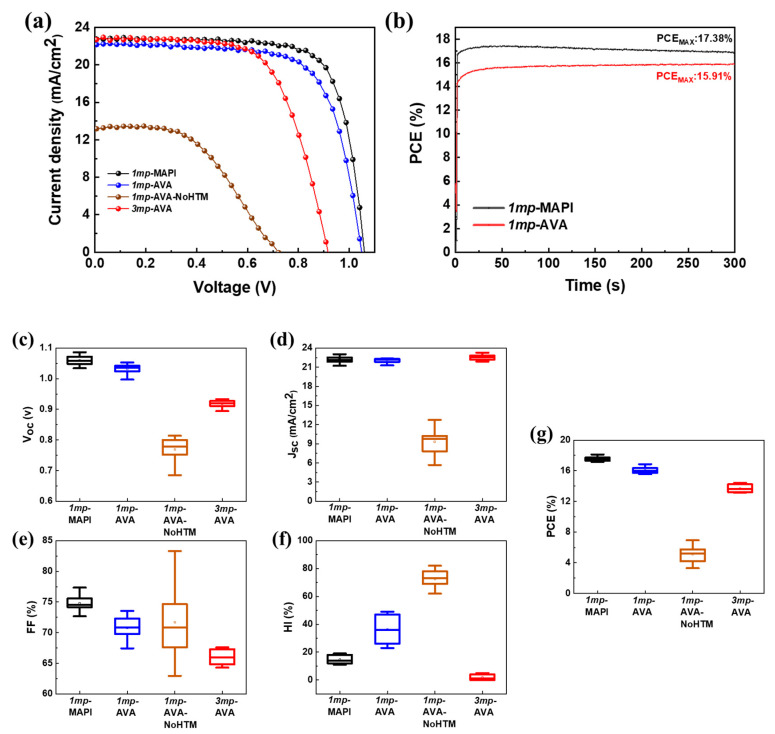
(**a**) Reverse scan *J-V* curves of *1mp*-MAPI, *1mp*-AVA, *1mp*-AVA-NoHTM, and *3mp*-AVA solar cells. (**b**) Tracking curves of *1mp*-MAPI and *1mp*-AVA best cells. (**c**–**g**) Statistical analysis of the solar cells *J-V* curve parameters: (**c**) *V_oc_*, (**d**) *J_sc_,* (**e**) FF, (**f**) HI and (**g**) PCE.

The *1mp*-MAPI, *1mp*-AVA, *1mp*-AVA-NoHTM, and *3mp*-AVA solar cells were characterized by measuring their current-voltage (*J-V*) curves. Table 1 gathers the results obtained for the best devices, while Figure 5a shows their reverse J-V curves, and Figure S3 (Supplementary Materials) shows the reverse and forward J-V curves. The *1mp*-MAPI cells exhibited the best performances. Their maximum PCE, measured on the reverse scan, was 18.09%. Their steady-state PCE, obtained by tracking at the potential of the maximum power output, *V_max_*, was found at 17.38% (Figure 5b). We observed that adding AVA was detrimental for the performances and the hysteresis of standard cells. The efficiency on the reverse scan decreased to about 16.86%. A large hysteresis was obtained in the presence of AVA. The PCE reduction was a consequence of lower *V_oc_*, *J_sc_*, and fill factor (FF). We also noted that the tracking curve showed a slow *1mp*-AVA cell response, but the stabilized PCE reached 15.91% (Figure 5b). In spite of the large hysteresis, this cell-stabilized PCE was close to the PCE value determined on the reverse scan. Our *1mp*-AVA cells were more efficient than those reported in the literature up to now [23]. Figure 5c shows the statistical analysis of the cells’ *J-V* curve parameters, which confirms the trend found for the best *1mp*-MAPI and *1mp*-AVA devices. The degraded performance of the *1mp*-AVA cells is due to the more defective morphology with pinholes. 

We also compared *1mp*-AVA cells with and without HTL (Table 1, Figure 5a,c). The efficiency dropped in the absence of HTL because many recombinations occur at the gold backelectrode when in direct contact with the perovskite layer. The efficiency was measured less than 7%; the *J-V* curve was S-shaped, and the hysteresis was very large. These cells presented the largest dispersion in their *J-V* curves (Figure 5c). Changing the cell structure and using the three mesoscopic ones dramatically changed the performances of the devices without HTM. In the case of *3mp* cells, with a carbon backcontact, the efficiency was much higher. The best cell achieved a PCE of 14.4% without significant hysteresis. For the *1mp*-AVA-NoHTM, the contact between perovskite and gold is poor. On the contrary, the mesoporous carbon layer can provide huge contact area for perovskite absorber, and thus assist the charge transfer. The absence of hysteresis can be assigned to the confined environment that would block the iodide ionic mobility [41]. Compared to the *1mp*-MAPI cell, the lower efficiency is mainly due to lower *V_oc_* and FF (Figure 5c). The external quantum efficiency (EQE) curves of the cells are disclosed in Figure S4 (Supplementary Material). A good correlation was found between the *J_sc_* calculated from the EQE spectra for the *1mp* cells. On the other hand, this parameter is significantly underestimated for the *3mp*-AVA cell due to its slow photoelectrical response.

### 3.3. Effect of Light Intensity at V_oc_

**Figure 6:**
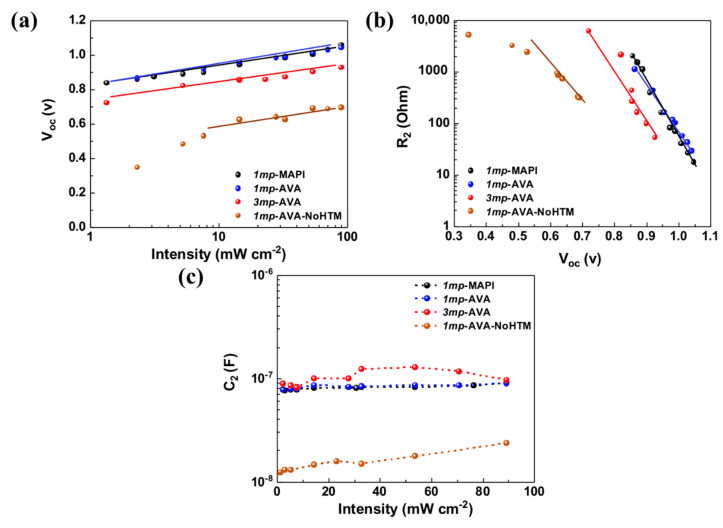
(**a**) Open-circuit voltage (*V_oc_*) versus light intensity (power density) curves. (**b**) Variation of R_2_ versus *V_oc_*. (**c**) C_2_ versus light intensity.

The effects of the AVA additive and cell architecture on the solar cells response to the light illumination at the open-circuit voltage (*V_oc_*) were investigated. Figure 6 shows the variation of this parameter with white light illumination power density (intensity). For the investigated cells, the *V_oc_* was superior with HTM compared to without HTM. Except for the *1mp*-AVA-NoHTM cell, the *V_oc_* scaled logarithmically with the light power density (*I*) and followed this relationship:

*qV_OC_* = *E_g_* + *n**_ID_**kT* ln(*I*/*I*_0_) (1)

with *E_g_* the bandgap, *q* as the elementary charge, *k* the Boltzmann constant, *T* the absolute temperature, and *n**_ID_*** the ideality factor. The latter parameter is related to the main recombination phenomena occurring at the *V_oc_* [34]. The values of *n_ID_* are gathered in Table 2. For *1mp*-cells with HTM and the *3mp-*cell, values between 1.8 and 2 are found, which suggest that, at the open circuit, a Shockley–Read–Hall (SRH) recombination mechanism through perovskite intragap defects is the dominating recombination process at *V_oc_* [34]. For the *1mp*-AVA-NoHTM cell, the points were not aligned, and Equation (1) was not valid at low light intensity. It shows a fast decrease of the *V_oc_* at low light intensity, and it also shows that strong recombination occurs.

The PSCs were further investigated by electrical impedance spectroscopy. The effect of light intensity on the impedance spectra at *V_oc_* is displayed in Figure S5 (Supplementary Materials). For the *1mp* cells with a HTL, the spectra and the behavior were close. They presented a large semicircle at high frequency and a second relaxation at low frequency. The former, which dominated the spectra, expended continuously with reducing the light intensity. The two other samples presented a different behavior. The *1mp*-AVA-NoHTM cells exhibited an additional relaxation at very high frequency, an inductive loop at intermediate frequency, and a second circle arc at low frequency. The *3mp*-AVA cells presented a spiral-like aspect at low frequency. 

In all cases, we focused the analysis on the high frequency circle arc. It was fitted by a R_2_//CPE_2_ circuit, except for the badly functioning *1mp*-AVA-NoHTM device for which a R_1_//CPE_1_ circuit in series with the R_2_//CPE_2_ one was employed to better fit the deviation from a circle arc (Figure S6a, Supplementary Materials). The former additional elements (R_1_ and CPE_1_) are discussed in the next section. CPE is a constant phase element from which a capacitance is extracted [20,42]. Details on the analysis of impedance spectra of perovskite solar cells can be found in the previous works by some of the authors in this present study [17,20,35,36,42]. We must state that, due to a different cell geometry and perovskite loading, in this section and in the next one (Section 3.4), the values of the extracted electrical elements cannot be compared between the *1mp* and *3mp* cells.

R_2_ is plotted as a function of the *V_oc_* in Figure 6b. The curves fitted an exponential function, from which we have determined an ideality factor, noted as *n’**_ID_***. In Table 2, we compare *n**_ID_***, which was determined from the *V_oc_*, and the *n’**_ID_*** extracted from the R_2_ curves. The two parameters are in good agreement and, more importantly, they vary in a similar manner with the cell type. We can conclude that R_2_ is a recombination resistance that measures the recombination in the bulk perovskite. We also extracted the high frequency capacitance C_2_ from the impedance spectra and reported this parameter in Figure 6c. For *1mp* cells with a HTM, they are similar and do not significantly vary with *V_oc_*. This capacitance is assigned to the bulk perovskite dielectric properties. The AVA did not significantly change this parameter. On the other hand, the deviation observed for *1mp*-AVA-NoHTM can be assigned to the presence of the inductive loop on the EIS spectra and to the additional R_1_//CPE_1_ circuit, which renders the accurate determination of C_2_ more difficult. In the case of *3mp*-AVA cells, the lower value of C_2_ for the *3mp*-AVA cell is related to a different architecture and device size.

### 3.4. Effect of Applied Potential on Impedance Spectra

We further investigated the effect of the AVA additive and cell architecture by studying the cells electrical responses at various applied voltage (*V_appl_*). Figure S7 (Supplementary Materials) shows the spectra. The two *1mp* cells with HTM (Figure S7a,b, Supplementary Materials) presented similar spectral features: a circle arc at high frequency, a second circle arc at low frequency, and, at its foot, a shoulder that was taken into account by using a R_3_//CPE_3_ electrical element in the equivalent electrical circuit. This circuit is presented in Figure S6b (Supplementary Materials). 

The *1mp*-AVA-NoHTM impedance spectra exhibited at high frequency the two relaxations described above (Figure S7c, Supplementary Materials). At intermediate frequency, a flat intermediate feature was found while a circle arc was present at low frequency. For the *3mp*-AVA cell, one could distinguish at low *V_appl_* at least four relaxations noted as I, II, III, and IV in Figure S7d (Supplementary Materials). At higher *V_appl_*, the shape described in the previous section with the spiral at low frequency was found. The analysis of the intermediate and low frequency electrical response of the *3mp* cell is not straightforward, and we focused our analysis on the high frequency arc of circle.

*R_s_* element values were determined from the extrapolation of the spectra at very high frequencies on the x-axis (impedance real part). This is due to the contacts and wire resistances. The higher *R_s_* for the *3mp*-AVA cell is assigned to the larger size of the cell and to the carbon backcontact, which is less conducting than the gold one. The *1mp*-AVA-NoHTM presented also a rather high *R_s_* and a high frequency R_1_//CPE_1_ component in spite of the use of a gold backcontact. It could be due to a layer formed at the interface between the PVK and gold, which would introduce an additional resistance and capacitance. The gold evaporation process causes damages on the PVK layer at the origin of the defects that act as recombination centers. This phenomenon is deleterious for photovoltaic properties.

**Figure 7:**
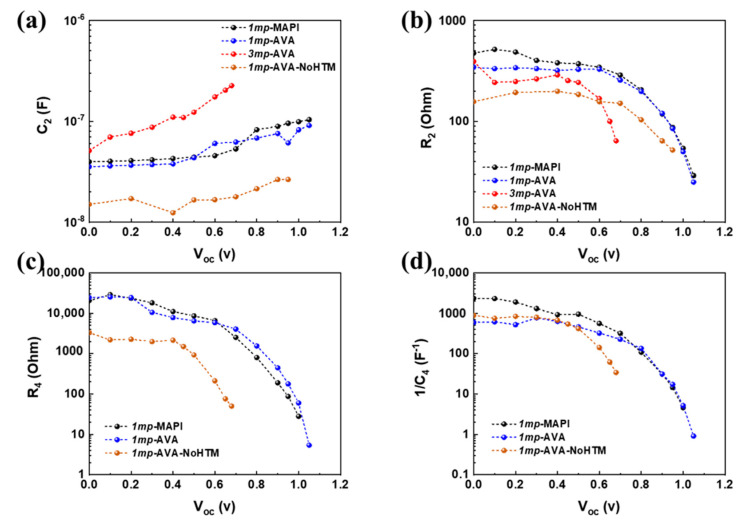
Effect of the applied voltage (*V_appl_*) on the solar cells electrical parameters: (**a**) C_2_, (**b**) R_2_, (**c**) R_4_ and (**d**) 1/C_4_.

C_2_ is presented in Figure 7a. The *1mp*-MAPI and *1mp*-AVA cells have similar behavior. At low applied voltage, C_2_ is assigned to the dielectric intrinsic capacitance of the PVK layer. Introducing AVA does not change the permittivity of the layer significantly. The fact that C_2_ is unchanged over a large *V_appl_* in the *3mp* device suggests that C_2_ is a dielectric intrinsic capacitance of MAPI-AVA material filling the pores. Higher and increasing C_2_ values are found for the *1mp*-AVA-NoHTM cell, which suggest several contributions for this electrical component.

R_2_ and R_4_ are analyzed as recombination resistances. The higher these resistances, the lower the charge recombinations, and the best is the cell [20,42]. In general, the AVA seems detrimental for these resistances. The XRD shows a slightly worse crystallinity, and the SEM views (Figure 3b) reveal the presence of pinholes in *1mp*-AVA layers, which are detrimental for the performances. The lower resistances values reflect these features. The voltage at which R_2_ and R_4_ parameters drop is linked to the cells *V_oc_*. It is quite close for *1mp*-MAPI and *1mp*-AVA cells. However, when HTM is absent in the *1mp* cell, these parameters drop at a low *V_appl_*, showing a recombination acceleration that results in the low *V_oc_* recorded. In our recent investigation [20], the origin of C_4_ is discussed. It was analyzed as a recombination capacitance related to the ion mobility that delays the charge recombination. C_4_ thus reflects the charge recombinations. Figure 7d shows that this parameter varies like R_2_ and R_4_, confirming this analysis. The *1mp*-AVA-NoHTM cell presents low 1/C_4_ above 0.5 V in good agreement with the large hysteresis of these cells and the low *V_oc_*.

We can also note that when an inductive loop is present, the total resistance, measured at low frequency, decreases more quickly than in the absence of this feature. A quick decrease of the total resistance leads to smaller *FF* on the *J-V* curves, which is then detrimental for the device performances. This is the case of *1mp*-AVA-NoHTM and *3mp* cells.

At this stage, based on the present knowledges of the scientific community on the impedance response of PSCs, we are unable to assign an origin and discuss the inductive loop as well as C_3_ and R_3_ elements. The latter were extracted from fitting, and the curves obtained are displayed in Figure S6 (Supplementary Materials).

## 4. Conclusions

In summary, we investigated the effect of an AVA additive and of the cell structure on PSCs. In the presence of an AVA, no 2D-perovskite phase was detected. AVA is deleterious for the PVK film morphology when the *1mp* substrate is used, while it is beneficial in reducing PVK bulk defects. Using a *3mp* scaffold leads to a significant improvement of the PVK structural quality as shown by TRPL and solves the potential problems of pinhole and related electrical shunt pathways. The *1mp*-AVA cells achieved a PCE of 16.9%, stabilized at 15.9%. This efficiency is significantly lower with higher hysteresis compared to *1mp*-MAPI cells. The ideality factor study shows recombinations via the SRH process at the open-circuit voltage. Our EIS study stated that the R_2_ element also varies with the *V_oc_*, like a recombination resistance from which we extracted an ideality factor comparable to the ones determined from the *V_oc_*. The EIS study at various applied voltages allowed us to extract two other parameters, R_4_ and C_4_, that were shown to be related to recombinations. Higher recombinations have been found in the case of the *1mp*-AVA cells compared to *1mp*-MAPI cells that are linked to the presence of pinholes in the PVK layer. We showed that the mesoporous carbon backelectrode functions differently to the gold one in the case of HTL-free devices. The PVK/gold interface presents defects and likely a damaged interlayer at the origin of poor performances. In the case of *3mp*-AVA cells, the intermediate frequency of EIS features, especially an inductive loop, is at the origin of a fast decrease of the total cell resistance with *V_appl_* and then to a low *FF* and *V_oc_* compared to the *1mp* cells with HTM. 

We also unveiled that MAPI is degraded by moisture with the formation of a monohydrate MAPI intermediate phase prior to PbI_2_ formation and MA release. The mechanism is irrespective of the PVK environment. AVA was shown to be beneficial for the stability of the MAPI layer. Moreover, a slower degradation was also found when MAPI-AVA PVK was embedded in the triple mesoscopic all-inorganic scaffold. 

## Figures and Tables

**Table 1:** PCE, J-V curve parameters, and HI of the best cells.

Cell Structure	PVK	Scan Direction	*V_oc_ (*V)	*J_sc_* (mA cm^−2^)	FF ^a^	PCE (%)	HI ^b^
*1mp*	MAPI	Reverse	1.058	22.78	75.03	18.09	0.12
		Forward	1.044	22.70	66.91	15.86	
*1mp*	AVA	Reverse	1.040	22.05	73.54	16.86	0.31
		Forward	1.017	21.94	51.87	11.58	
*1mp*	AVA-NoHTM	Reverse	0.805	12.75	67.62	6.94	0.73
		Forward	0.790	6.71	35.56	1.89	
*3mp*	AVA	Reverse	0.932	22.88	67.62	14.41	0.00
		Forward	0.929	22.86	68.23	14.49	

^a^ Fill factor. ^b^ Hysteresis index defined as: [PCE(%)_rev_ − PCE(%)_for_]/PCE(%)_rev_.

**Table 2:** Ideality factors obtained from open-circuit potential (*n****_ID_***) and EIS (R_2_) (*n’****_ID_***) measurements for the four different PSCs.

	*n_ID_*	*n’_ID_*
*1mp*-MAPI	1.99	1.56
*1mp*-AVA	1.89	1.92
*1mp* AVA-NoHTM ^a^	3.50	3.44
*3mp*-AVA	1.82	1.64

^a^ Fit over the whole intensity and *V_oc_* range.

## Supplementary Materials

The following are available online at https://www.mdpi.com/2079-4991/10/12/2512/s1. Figure S1: (a) TRPL of *1mp*-MAPI and *1mp*-AVA layers deposited on glass. (b) TRPL of *3mp*-MAPI and *3mp*-AVA layers deposited on mesoporous ZrO_2_/glass. Figure S2: Pictures of PVK layers: fresh and after 20 h and 120 h aging at ≥90% RH/RT. The black/grey aspect of the *3mp* samples is due to the carbon back electrode. (a) *1mp*-MAPI, (b) *1mp*-AVA, (c) *3mp*-MAPI and (d) *3mp*-AVA; Figure S3: Forward and reverse *J-V* curves of (a) *1mp*-MAPI, (b) *1mp*-AVA, (c) *1mp*-MAPI-NoHTM and (d) *3mp*-AVA best cells; Figure S4 External quantum efficiency, EQE, spectra and *J_sc_* integration curves of the various cells; Figure S5: Effect of the light intensity in sun% on the impedance spectra measured at the *Voc*. (a) *1mp-MAPI*, (b) *1mp-AVA*, (c) *1mp-AVA-NoHTM* and (d) *3mp-AVA*.; Figure S6: (a,b) Equivalent electrical circuits employed to fit impedance spectra of PSCs. See the core text for explanation. R_s_, R_1_, R_2_, R_3_ and R_4_ are resistances. CPE_1_, CPE_2_, CPE_3_ and CPE_4_ are constant phase elements; Figure S7: Effect of the applied voltage on the impedance spectra of the investigated cells. (a) *1mp*-MAPI, (b) *1mp*-AVA (c) *1mp*-AVA-NoHTM, (d) *3mp*-AVA low voltage and (e) *3mp*-AVA high voltage; Figure S8: Effect of *V_appl_* on (a) *R_s_*, (b) R_3_ and (c) C_3_ parameters. Table S1: Fitting parameters by a triple exponential function of the TRPL curves of Figure S1.
