# Supplementary material for: Effects of 5-Ammonium Valeric Acid Iodide as Additive on Methyl Ammonium Lead Iodide Perovskite Solar Cells
Source: Nanomaterials (Basel). 2020 Dec 14;10(12):2512. doi: 10.3390/nano10122512 (PMC7765157; doi:10.3390/nano10122512)
Supplement: Supplementary file 1 [file nanomaterials-10-02512-s001.pdf]

nanomaterials

Supplementary Materials

Effects of 5-Ammonium Valeric Acid Iodide as Additive on Methyl Ammonium Lead Iodide Perovskite Solar Cells

Daming Zheng [1], Changheng Tong [1,2], Tao Zhu [1], Yaoguang Rong [2] and Thierry Pauporté [1,*]

1 Chimie ParisTech, PSL Research University, CNRS, Institut de Recherche de Chimie Paris (IRCP), UMR8247, 11 rue P. et M. Curie, F-75005 Paris, France; daming.zheng@chimieparistech.psl.eu (D.Z.); tch13986581570@gmail.com (C.T.); tao.zhu@chimieparistech.psl.eu (T.Z.)

2 Wuhan National Laboratory for Optoelectronics, China-EU Institute for Clean and Renewable Energy, Huazhong University of Science & Technology (HUST), 430074, Hubei, China; ygrong@hust.edu.cn

* Correspondence: thierry.pauporte@chimieparistech.psl.eu

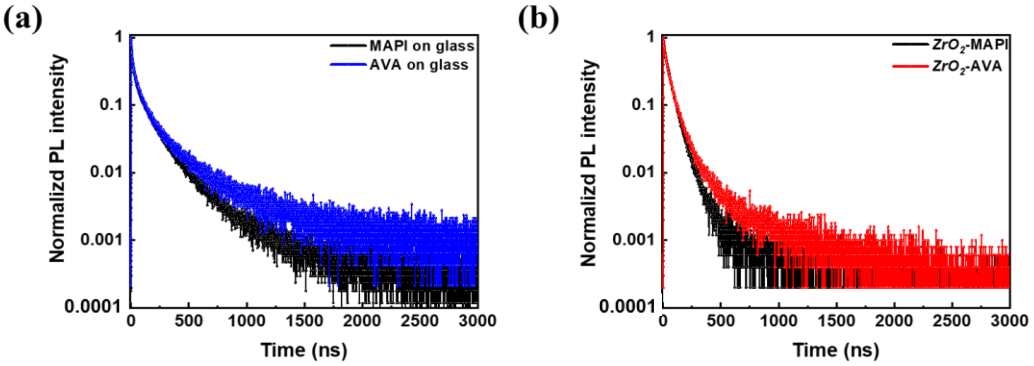

Figure S1. (**a**) TRPL of *1mp*-MAPI and *1mp*-AVA layers deposited on glass. (**b**) TRPL of *3mp*-MAPI and *3mp*-AVA layers deposited on mesoporous ZrO_2/glass.

Table S1. Fitting parameters by a triple exponential function of the TRPL curves of Figure S1.

	**Fitting function:** $y = A_1\exp(-x/\tau_1) + A_2\exp(-x/\tau_2) + A_3\exp(-x/\tau_3) + y_0$									
	y_0	A_1	τ_{fast} (ns)	RC_{fast} [a]	A_2	τ_{int} (ns)	RC_{int} [a]	A_3	τ_{slow} (ns)	RC_{slow} [a]
MAPI on glass	7.6E-4	0.39	3.21	0.03	0.40	25.17	0.25	0.19	153.51	0.72
AVA on glass	1.6E-3	0.452	5.31	0.05	0.39	42.14	0.36	0.12	227.45	0.59
MAPI on ZrO_2/Glass	3.8E-4	0.31	6.93	0.05	0.58	39.31	0.49	0.24	88.01	0.46
AVA on ZrO_2/Glass	5.6E-4	0.41	9.85	0.09	0.61	51.47	0.65	0.07	181.4	0.26

[a] Relative contribution

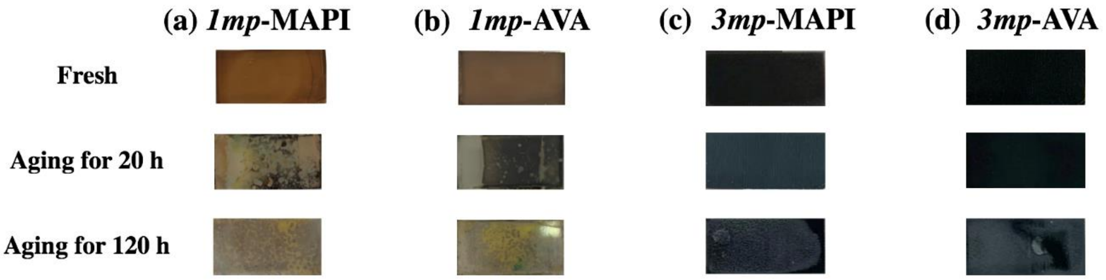

Figure S2. Pictures of PVK layers: fresh and after 20 h and 120 h aging at ≥90% RH/RT. The black/grey aspect of the *3mp* samples is due to the carbon back electrode. (**a**) *1mp*-MAPI, (**b**) *1mp*-AVA, (**c**) *3mp*-MAPI and (**d**) *3mp*-AVA.

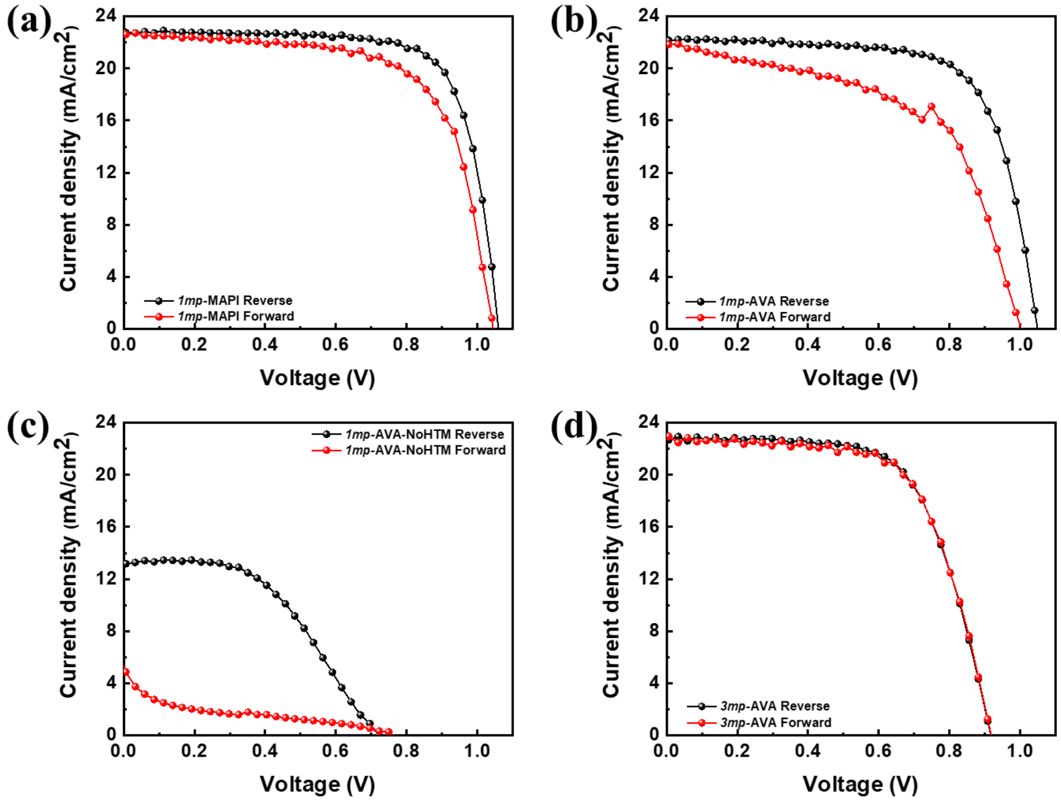

Figure S3. Forward and reverse *J-V* curves of (**a**) *1mp*-MAPI, (**b**) *1mp*-AVA, (**c**) *1mp*-MAPI-NoHTM and (**d**) *3mp*-AVA best cells.

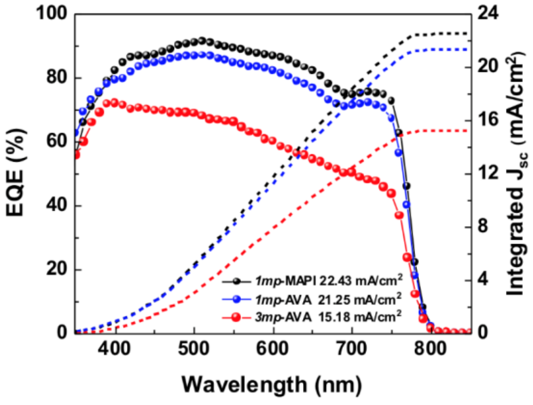

Figure S4. External quantum efficiency, EQE, spectra and J_{sc} integration curves of the various cells.

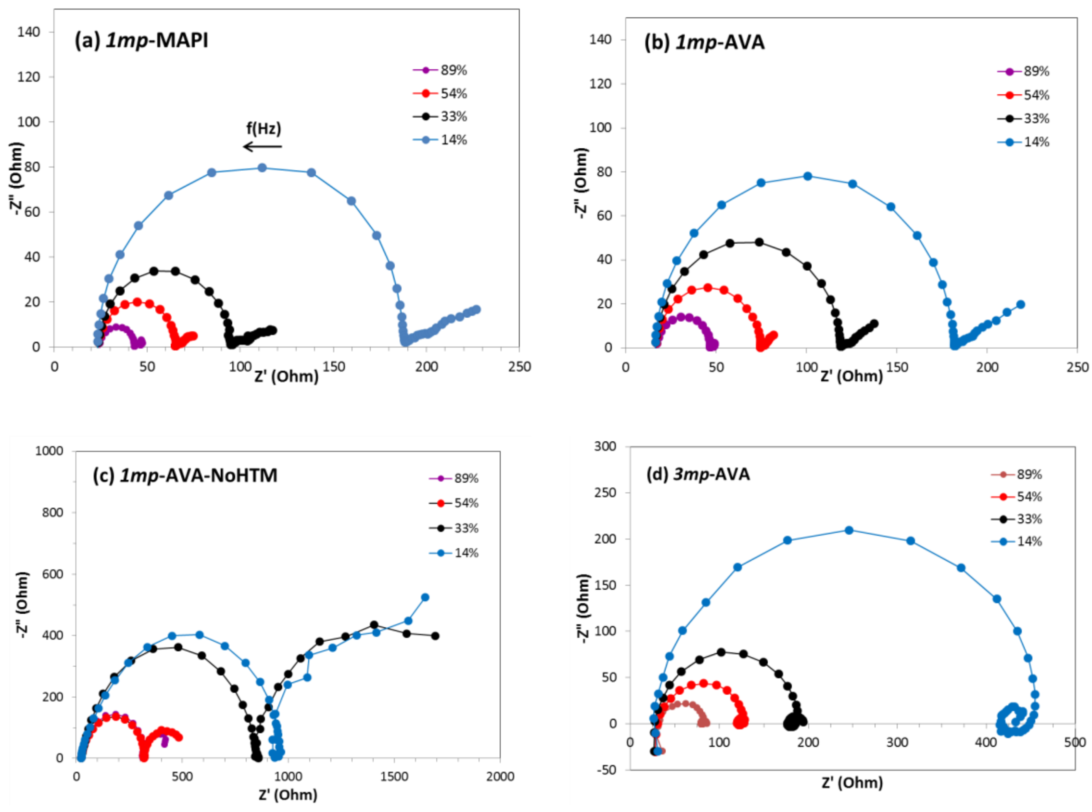

Figure S5. Effect of the light intensity in sun% on the impedance spectra measured at the V_{oc}. (**a**) *1mp*-MAPI, (**b**) *1mp*-AVA, (**c**) *1mp*-AVA-NoHTM and (**d**) *3mp*-AVA.

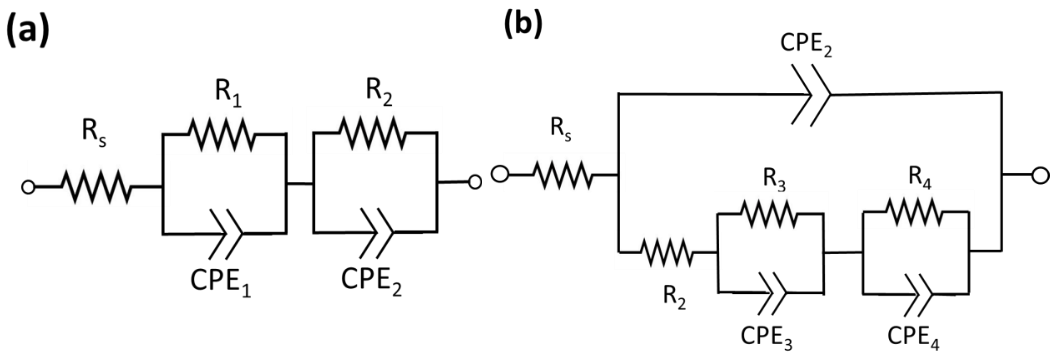

Figure S6. (**a**,**b**) Equivalent electrical circuits employed to fit impedance spectra of PSCs. See the core text for explanation. R_s, R_1, R_2, R_3 and R_4 are resistances. CPE_1, CPE_2, CPE_3 and CPE_4 are constant phase elements.

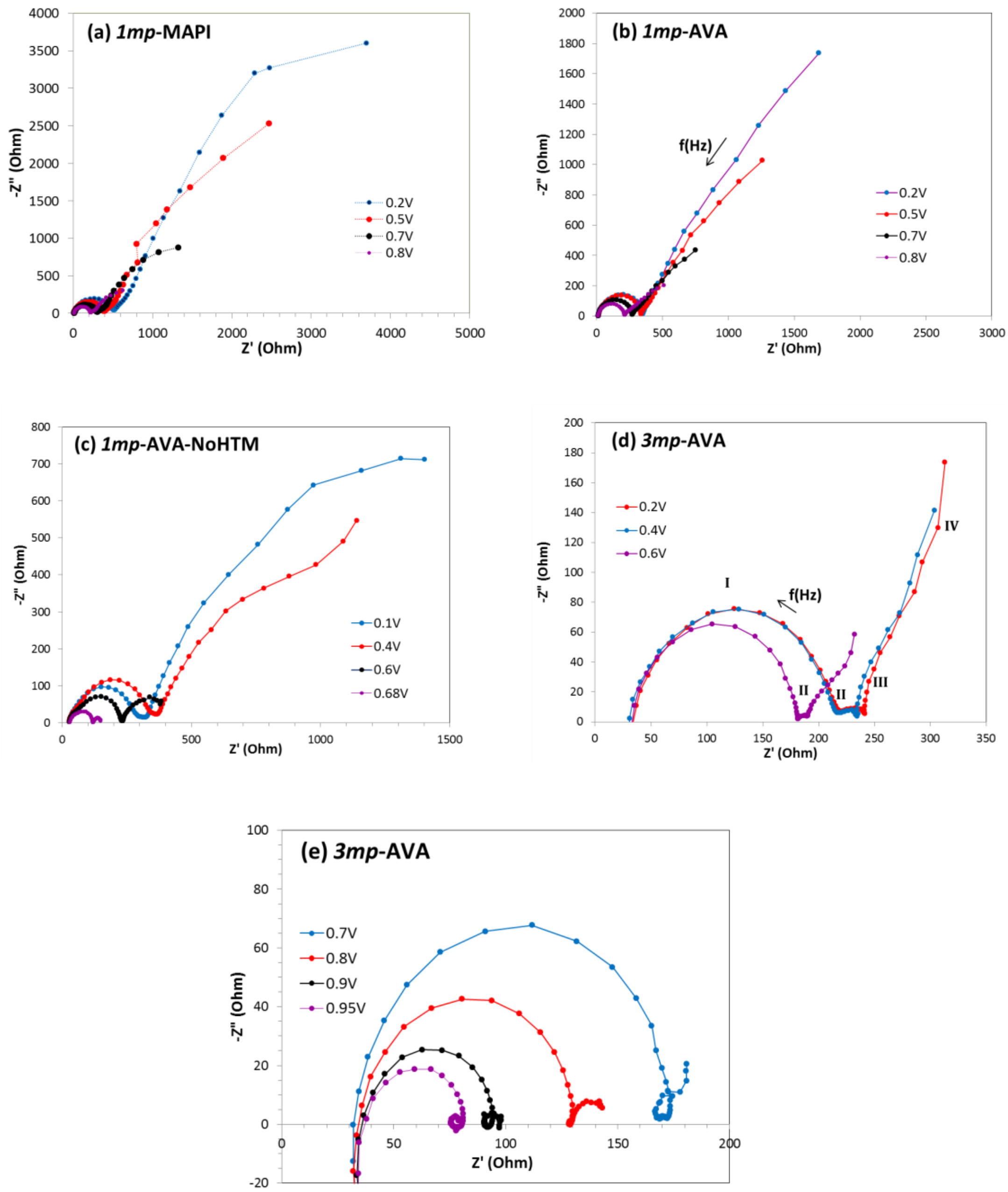

Figure S7. Effect of the applied voltage on the impedance spectra of the investigated cells. (a) *1mp*-MAPI, (b) *1mp*-AVA (c) *1mp*-AVA-NoHTM, (d) *3mp*-AVA low voltage and (e) *3mp*-AVA high voltage.

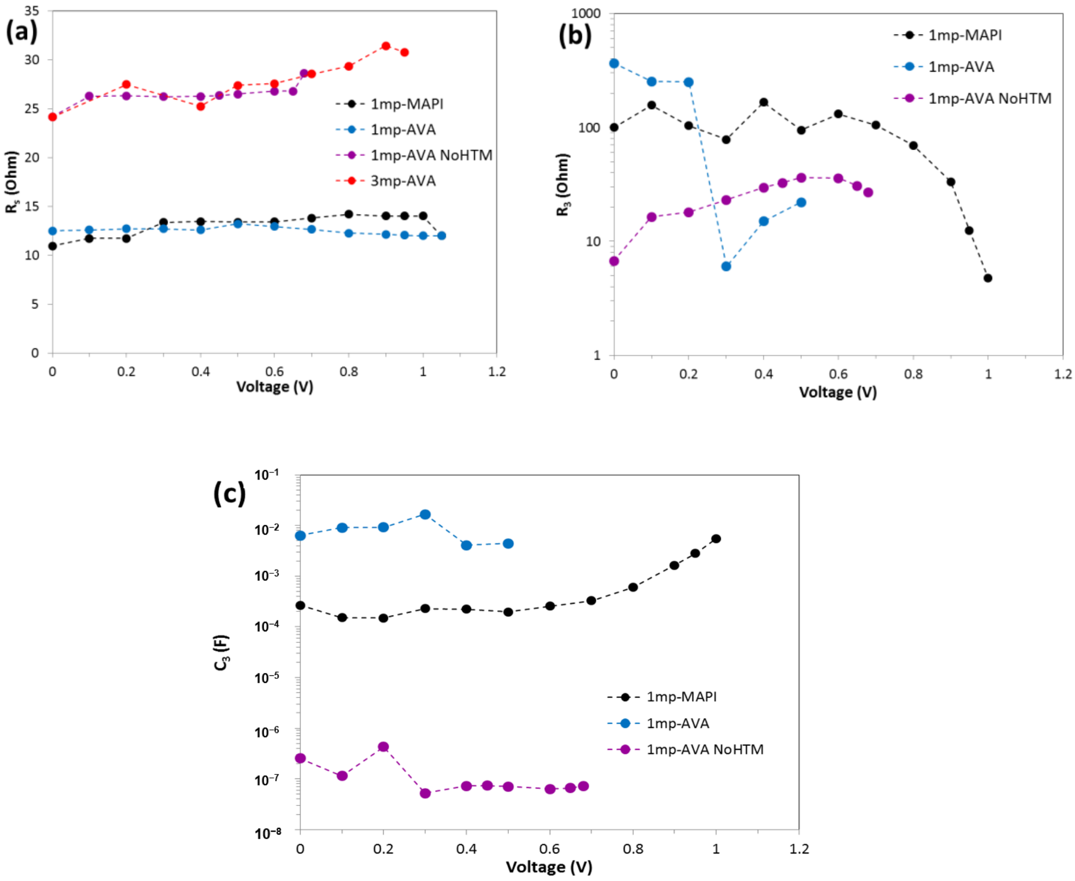

Figure S8. Effect of V_{appl} on (**a**) R_s, (**b**) R_3 and (**c**) C_3 parameters.

Publisher's Note: MDPI stays neutral with regard to jurisdictional claims in published maps and institutional affiliations.

© 2020 by the authors. Licensee MDPI, Basel, Switzerland. This article is an open access article distributed under the terms and conditions of the Creative Commons Attribution (CC BY) license (http://creativecommons.org/licenses/by/4.0/).
